# Can Studying Genetically Predisposed Individuals Inform Prevention Strategies for RA?

**DOI:** 10.3390/healthcare9101301

**Published:** 2021-09-29

**Authors:** Amanda Fowler-Woods, Irene Smolik, Vidyanand Anaparti, Liam O’Neil, Hani El-Gabalawy

**Affiliations:** 1 Ongomiizwin Indigenous Institute of Health and Healing, Rady Faculty of Health Sciences, University of Manitoba, Winnipeg, MB R3W 0W3, Canada; umwoodsa@myumanitoba.ca; 2 Rheumatic Diseases Unit, Department of Internal Medicine, Rady Faculty of Health Sciences, University of Manitoba, Winnipeg, MB R3A 1M4, Canada; irene.smolik@umanitoba.ca; 3 Manitoba Center for Proteomics and Systems Biology, Department of Internal Medicine, Rady Faculty of Health Sciences, University of Manitoba, Winnipeg, MB R3E 3P4, Canada; vidyanand.anaparti@umanitoba.ca (V.A.); liam.oneil@umanitoba.ca (L.O.)

**Keywords:** rheumatoid arthritis, autoimmune disorder, prevention, risk, Indigenous population, First Nations, first-degree relatives, randomized clinical trials

## Abstract

Rheumatoid arthritis (RA) is a prevalent autoimmune disorder in which complex genetic predisposition interacts with multiple environmental factors to precipitate chronic and progressive immune-mediated joint inflammation. Currently, in most affected individuals, ongoing suppression of the inflammation is required to prevent irreversible damage and functional loss. The delineation of a protracted preclinical period in which autoimmunity is initially established and then evolves to become pathogenic provides unprecedented opportunities for interventions that have the potential to prevent the onset of this lifelong disease. Clinical trials aimed at assessing the impact of specific prevention strategies require the identification of individuals who are at high risk of future RA development. Currently, these risk factors include a strong family history of RA, and the detection of circulating RA-associated autoantibodies, particularly anti-citrullinated protein antibodies (ACPA). Yet, even in such individuals, there remains considerable uncertainty about the likelihood and the timeframe for future disease development. Thus, individuals who are approached to participate in such clinical trials are left weighing the risks and benefits of the prevention measures, while having large gaps in our current understanding. To address this challenge, we have undertaken longitudinal studies of the family members of Indigenous North American RA patients, this population being known to have a high prevalence of RA, early age of onset, and familial clustering of cases. Our studies have indicated that the concepts of “risk” and “prevention” need to be communicated in a culturally relevant manner, and proposed prevention interventions need to have an appropriate balance of effectiveness, safety, convenience, and cultural acceptability. We have focused our proposed prevention studies on immunomodulatory/anti-inflammatory nutritional supplements that appear to strike such a complex balance.

## 1. Genetic Risk Factors for RA

It is estimated that genetic factors account for almost half of the risk of rheumatoid arthritis (RA) development, based on studies of families and monozygotic/dizygotic twins (reviewed in [1]). In recent decades, numerous studies in populations worldwide have incrementally demonstrated multiple genes that contribute to the genetic risk for RA, with the recent studies suggesting that more than one hundred genes contribute significantly, albeit mostly with low odds ratios [2,3]. It should be noted that the cumulative genetic risk based on these existing genetic associations, identified using methods such as genome-wide association studies (GWAS), continues to fall substantially below the estimated ~50% heritability of this disease, a phenomenon that has been called “missing heritability”. This discrepancy may partially relate to the finding that a substantial proportion of the genetic risk may be found in regulatory genomic segments rather than the expressed genome.

Of the multiple genetic associations that have been demonstrated, the largest contribution to RA risk is from the MHC, specifically from the HLA-DRB1 locus, which has most recently been estimated to represent ~11% of the total genetic variance [3]. Specific HLA-DRB1 alleles are known to be associated with RA, but the prevalence of these alleles differs substantially worldwide. For example, HLA-DRB1*0401 is a risk allele demonstrated primarily in Caucasian populations, while HLA-DRB1*0405 is a risk allele in Japanese populations, and HLA-DRB1*1402 is a risk allele seen almost exclusively in Indigenous North American (INA) populations. To date, the best explanation for these heterogeneous HLA associations is the shared epitope (SE) hypothesis first proposed by Gregersen and his colleagues more than 30 years ago [4]. This hypothesis proposes that a positively charged amino acid sequence in position 70–74 of the HLA-DRB1 molecule is critical for imparting the RA genetic risk. Since it was first published in 1987, there have been several refinements to the SE hypothesis, including a more recent analysis suggesting that amino acids in other parts of the molecule, particularly positions 11 and 13, are also quite important for imparting RA risk [5]. Further insights regarding the mechanism by which the positively charged QKRAA, QQRAA, and KKRAA SE sequences increase the risk of RA has come from studies demonstrating that the peptide-binding P4 pocket of the HLA-DRB1 molecule, for which the SE sequence forms the sidewall, presents citrullinated antigens efficiently to T cells [6]. This critical observation serves to mechanistically link the HLA genetic risk to the anti-citrullinated protein antibody [ACPA] response that is now known to be the hallmark of seropositive RA.

Evolving technologies are rapidly providing key insights into how the genomic risk factors impact on specific pathways that are involved in the pathogenesis of RA. For example, the PTPN22 locus is strongly associated with the risk of RA and other autoimmune diseases [7,8]. This locus encodes for a tyrosine phosphatase that regulates the activation of lymphocytes, and a specific mutation in this gene is associated with increased T and B cell responsiveness. Other examples of how RA-associated genetic risk factors may mediate this increased risk include PADI4 which regulates citrullination of proteins [9] as well as STAT4 and REL, both of which regulate cytokine signal transduction [10,11].

Yet, despite a dramatic increase in our understanding of how genetic predisposition results in the subtle and complex molecular changes that ultimately lead to RA clinical manifestations, the clinical utility of testing for these genetic risk factors remains uncertain. For example, studies that have attempted to develop composite genetic risk scores (GRS) that can be used in conjunction with other prognostic factors have largely found that the inclusion of the GRS adds only modestly to the models, although the genetic risk for seropositive and seronegative RA differed [12]. There remains a key gap regarding how population-based genetic associations can be used at an individual level. Although some progress has been made in terms of RA pharmacogenomics, this approach continues to be in its early stages.

**Figure 1:**
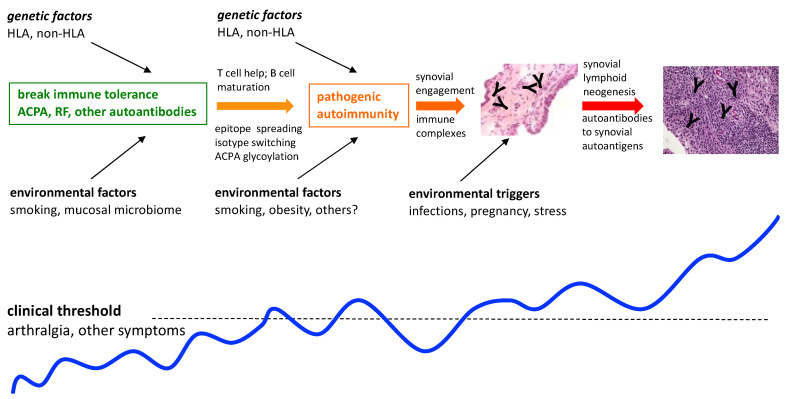
Preclinical stages toward RA development.

Based on multiple studies indicating that there is a prolonged preclinical stage for RA that is characterized by the presence of circulating RA autoantibodies such as ACPA and RF, in otherwise clinically unaffected individuals, illustrated in Figure 1, there are now several international initiatives aimed at preventing or delaying the onset of the disease [13,14]. In the context of such emerging prevention approaches, we need to carefully consider the following: how does knowledge of genetic risk help us, and what is the importance and impact of having a strong family history of RA on the perception of risk, willingness to participate in the trials, and the ultimate outcomes of the trials? These questions encompass a spectrum of biological, ethical, sociological, and cultural considerations that are inextricably intertwined. In the following paragraphs, we will attempt to explore a number of these considerations. We will focus on Indigenous peoples whom we have engaged with, these being predisposed on a population and on a familial level, to illustrate the complex intersection of these considerations. Some aspects are unique to these populations, while others are more generalizable to other predisposed populations.

## 2. Studies of RA Risk in Predisposed Indigenous North Americans

**Figure 2:**
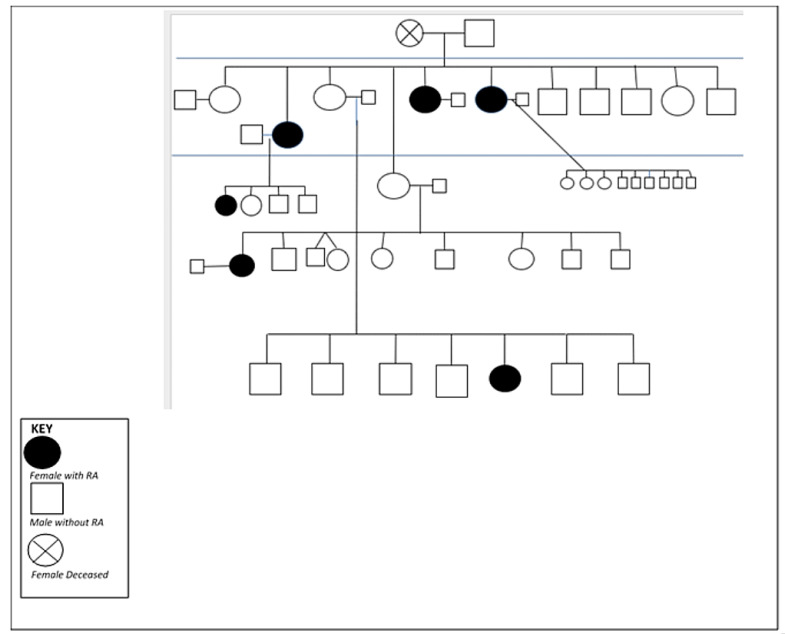
An example of an RA multi-case family pedigree.

Multiple studies have shown that the prevalence of RA is high in many, but not all, INA populations (summarized in [15,16]). We have studied RA in the Cree, Ojibwe, Oji-Cree peoples (collectively referred to as First Nations peoples) of Central Canada, and the Tlingit people of Alaska. Based on analysis of administrative data from Manitoba, Canada, it is estimated that the First Nations peoples of Central Canada have a 2–3-fold higher prevalence of RA compared to non-First Nations populations [17]. As shown in Figure 2, there is considerable familial clustering of this (and other) autoimmune disease(s) in the Indigenous populations we have studied [18]. Moreover, there is an early age of onset [19], inconsistent use of disease-modifying anti-rheumatic drugs (DMARD), and unfavorable outcomes such as progressive disability and premature mortality [20,21]. These observations suggested to us that genetic factors likely play a particularly important role in the risk of developing RA in this population, although it has been difficult to disentangle the effects of shared environments, access to specialized care, and other non-genetic considerations. Based on this, we established a prospective cohort of the first-degree relatives (FDR) of RA patients from this population and followed this cohort longitudinally for the past 15 years to obtain a better understanding of RA development in this predisposed population.

Since 2005, our team has worked in partnership with several First Nations communities and followed more than 750 unaffected family members of RA patients to determine their risk of developing future RA. Our research has compelled us to further examine prediction and prevention of future RA within this population. We therefore proposed a randomized clinical trial (RCT) to examine the effectiveness of a targeted dietary supplementation strategy to reduce risk of RA development. As a central component of this study, we also hope to understand how First Nations peoples view clinical trials that aim to prevent disease.

## 3. Participation of First Nations Peoples in RCTs

Understanding the impact and/or experience of participating in RCTs for Indigenous peoples, individuals, communities, and nations is an area of research that is continuing to develop. Researchers have reported on the participation of Indigenous peoples in RCTs, as well as some barriers and facilitators to this type of research. However, what is missing in the literature is a discussion of how the experience of participation, as well as the outcomes of such research, impacts Indigenous participants, communities, and nations within which the research takes place [22,23]. The scant literature that exists regarding RCTs specifically undertaken within Indigenous populations is primarily a description of the research or RCT itself [24,25,26,27].

While Indigenous peoples have been involved as participants within RCTs, they remain underrepresented considering the breadth and seriousness of health disparities experiences by Indigenous peoples [22,23,28,29]. This underrepresentation of Indigenous populations in RCTs leads to serious implications for clinical health research. Without being able to act on clinical research data generated from working with Indigenous populations specifically, there are limited opportunities for validation, generalizability and transferability of knowledge from these studies to Indigenous populations, barriers to understanding the evidence of health-based interventions within Indigenous populations, an inability to identify differences in the effectiveness of interventions and/or treatments between Indigenous and non-Indigenous people, and further marginalization of Indigenous peoples from knowledge about health based interventions [22,23]. Therefore, this underrepresentation in RCTs prevents the ability to establish a deeper understanding of the health disparities that impact Indigenous peoples, as well as the ideal interventions for this population [22,23]. As a result, there are still gaps in knowledge regarding the optimal condition of RCTs within Indigenous populations [22,23,30].

Stemming from this knowledge gap, RCTs are typically undertaken in non-Indigenous populations, and it is assumed that the results of these studies would be readily applicable to Indigenous peoples [23,28]. However, it must be acknowledged that there are numerous sociocultural contexts within Indigenous populations that create unique environments and ongoing health disparities and must be fully considered during the research process. Historical and contemporary contexts, including the ongoing damages of settler colonialism can create significant barriers to direct application of RCT findings and processes onto Indigenous populations [23,28].

## 4. Historical Context of Indigenous Health Research

It is important to consider that for many Indigenous people, perceptions of RCTs have been negatively skewed by a long history of unethical research practices and experiences of deception and harm, leading to an association between research and a failure to improve Indigenous health [22,28]. This has led to a widespread hesitancy or refusal to participate in research for many Indigenous peoples. Through much collaborative and dedicated work, Indigenous and non-Indigenous leadership have since worked to establish requirements that within Indigenous health research, consultation with and inclusion of Indigenous voices and perspective is now driving the expectations of research engagement with Indigenous peoples. It is now understood that the central most aspect of Indigenous health research is the consultation and inclusion of Indigenous peoples in the research design and process [22,23,25].

Indigenous health research can no longer hold priority to serve the needs of the researcher. Research must be based on the needs and interests of the Indigenous peoples, and direct research benefits must be meaningful and sustainable for the individuals, communities, and nations involved [22,23,28,31,32]. Researchers are also required learn about and understand the history and culture of the people and communities with which they choose to engage in order to build and sustain meaningful research relationships and partnerships [22,28]. Most importantly, the research and intervention must align with the cultural perspectives, practices, and beliefs of the community [22,23,33]. All Indigenous health research must adhere to these ethical guidelines inclusive of research that is respectful, useful, meaningful, relevant, and beneficial to the cultures, experiences, and lives of Indigenous peoples [22].

## 5. Guidelines for RCTs within Indigenous Populations

Researchers have suggested that it is imperative that national guidelines and methodological processes for conducting RCTs with Indigenous peoples be developed in order to have clearer directives and expectations for all those involved [22]. There are national and international ethical guidelines, frameworks, and principles for research engagement with Indigenous peoples which are useful. However, little is known their ability to address issues related to the use of RCTs with Indigenous populations [22]. In Canada, the large national funding agencies have developed a guidance framework for undertaking research with Indigenous peoples [32]. A framework for research with First Nations, Metis, and Inuit peoples also guides research within the Rady Faculty of Health Sciences at the University of Manitoba [34]. In addition, it is recommended that researchers working with Indigenous peoples, and in particular First Nations peoples, learn and follow the principles of Ownership, Control, Access, and Possession (OCAP) as well as recognize the United Nations Declaration on the Rights of Indigenous Peoples (UNDRIP) as the framework for reconciliation with Indigenous peoples in Canada [35,36]. Highlights of these frameworks, principles, and statements clearly articulate the need for respectful engagement with Indigenous peoples throughout all aspects of the research process, cultural appropriateness of all components of the research, for the generation of direct and meaningful benefits of the research for all Indigenous peoples and communities involved, and for the appropriate and community accepted models of data ownership and governance. Research focusing on the prevention prevalent chronic diseases such as RA is no exception to these guiding principles. As such, the study of “prevention” and “risk” need to be explored more fully within this context.

## 6. Prevention and Understanding “Risk”

The notion of family connectedness in First Nations communities was important for understanding the First Nations perspective of RA and risk, as the understanding and implications of illness and disease often centered around the family collective. Illness and disease impact the individual, but it is also impactful on the family and community. First Nations understanding of wellbeing occurs through a collective worldview where everything is based on relationships and how we react and exist within those relationships What happens to individuals also has impacts on families, communities, and nations [37]. In understanding this worldview, it became apparent that early detection is at a family and not only an individual level.

There are important ethical considerations to be addressed when discussing risk of disease, be it in non-First Nations or First Nations populations. In the context of First Nations, assessing and communicating RA risk based on the presence of autoantibodies or on genetic risk factors, involves unique cultural considerations, and is not simply based on statistical paradigms. Previous discussions between First Nations community members and our research group found that most of the family members of individuals with RA would be willing to participate in screening protocols that aim to define an individual’s risk of future disease development, but they want to be told the results of screening tests, irrespective of whether they were “positive” or not. Through this discussion, we learned that relatives of First Nations RA patients expressed interest in participating in this type of research based on a desire to help other First Nations peoples collectively rather than self-interest in their own health.

In roundtable focus groups with RA patients and their close relatives [mostly first-degree], it became clear that relatives had a different understanding of RA risk compared to that of the researchers who were planning to undertake prevention studies based on screening protocols. For example, there was a common understanding, passed down through generations of family members that all forms of “arthritis” resulted from working in wet, cold, outdoor environments such as trapping on the land. In fact, the Oji-Cree term arthrisit otaanikookanaanik ohkanaahpine can be translated to mean wet cold environments. It is thus crucial to acknowledge that cultural understanding is embedded in language, and adequate time and effort is needed to gain an understanding of these ways of knowing. Defining RA risk based on blood tests and not on these culturally determined “causes” required additional time and space for communication and discussion. Thus, communication of disease risk to Indigenous people must be done with thoughtful consideration of the historical and contemporary contexts of the communities and populations in which the risk is being communicated. There cannot be a one-size-fits-all approach to Indigenous research activities [33].

## 7. Multiple Ways of Knowing

It is important to note that differences between Western-based scientific views and First Nations perspectives regarding the “origins” of a disease such as RA are not due to lack of “knowing” by the latter. Rather, it should be acknowledged that it takes time to reconcile these different ways of knowing. In looking at differences in language it becomes apparent that western clinical understanding of RA disease does not translate adequately, as some scientific terms and explanations cannot be translated into Indigenous languages. Thus, different views occur in the context of an inability to translate directly between languages, and added time, effort, and resources are required to navigate cultural understandings. If one wants to understand prevention activities acceptable to First Nations communities, it important to first discuss what constitutes RA, how RA is diagnosed, how it differs from other non-autoimmune forms of arthritis such as osteoarthritis. A key component in this dialogue involves the individuals with RA in the community, particularly the close family relatives who are affected. Their experiential ways of knowing are indispensable in providing the needed background to begin to understand that rationale for estimating risk and why prevention strategies are even needed … why not just treat the disease when it starts?

In our focus group discussions, themes emerged that shed light on “prevention hesitancy”. This included fear of medication side-effects and a general lack of interest in lifestyle changes such as smoking cessation and dietary modifications. Convenience of any proposed prevention intervention was critical to those unaffected but felt to be “at-risk”. There was a desire to embrace an RA “vaccine” when it became available. Another key theme regarding potential participation was how the prevention study was presented and by whom. In the First Nations context, both individual and community consent are important for such studies. Interestingly, as alluded to above, participation in clinical trials of drug therapies to prevent RA were generally found to be acceptable … “if this will help our People”.

## 8. Diet and Nutrition Prevention Research

Identifying participation-related barriers and implementing strategies to address these gaps is critical for improving study recruitment in RA prevention trials [38,39]. While a negative view on the trial drug along with the fear of using medications remains an important barrier, factors such as reluctance toward adopting healthy lifestyle changes such as smoking cessation necessitate the need for looking at alternative strategies for future trials that can be better integrated with cultural habits of the communities [40]. Dietary interventions using natural remedies such as dietary supplements along with strategies such as personalized risk-estimation tools and optimal education on perceived disease risk seem to be promising facilitators that can potentially encourage trial participation and are well accepted [39,41,42].

The use of dietary supplements is highly prevalent in individuals with arthritis including those with RA, since people perceive them to provide a higher degree of self-control over their health [43,44]. However, evidence pertaining to the efficacy, and safety of these supplements on RA symptoms is conflicting and of moderate strength [45,46]. Randomized controlled trials on moderate-to-established RA patients provide evidence that diet can influence patient-reported outcomes including RA symptoms, disease activity score (DAS), arthralgia, and pain, after consuming certain dietary supplements or removing them from the diet [47,48]. Foods such as red meat, eggplant, tomato, beer, desserts, sugar-sweetened beverages, and soda were associated with worsening RA symptoms. On the other hand, interventions such as a Mediterranean diet (containing olive oil, fish, nuts, legumes, berries, and whole grains), spices (such as ginger, curcumin, or cinnamon), probiotics or supplements such as omega-3 fatty acids, and vitamin D were found to either improve disease symptoms, and/or reduce DAS28 and CRP scores [48,49,50]. Furthermore, long-term adherence to a Mediterranean or vegetarian diet significantly reduced the incidence rates of seropositive RA and mitigated development of anti-CCP antibodies in at-risk individuals, thereby highlighting the role of diet not only in patients with established disease but also in preclinical RA [30,31,46,49,51,52,53,54].

Beneficial effect of dietary components primarily stems from their ability to modulate inflammation either through having a direct effect on the underlying pathways that regulate disease processes or by restoring gut dysbiosis. Anti-inflammatory effects of dietary supplements, either individually or when used in combination, are manifold as we showed previously in a murine model of collagen-induced arthritis [55]. In animals with arthritis, curcumin alone or in combination with vitamin D and omega-3 fatty acids significantly reduced disease severity and suppressed cartilage degradation, apart from inhibiting the expression of multiple inflammatory proteins including cytokines, chemokines, and matrix metalloproteinases [55]. Diet also shapes the diversity and composition of the gut microbiome, which in turn primes the mucosal immune system, maintains immune homeostasis in the host and regulates gut permeability and integrity of the intestinal barrier. Diversity of the gut microbiome is significantly altered both in seropositive FDR and RA patients and is characterized by significant enrichment of butyrate-producing bacteria such as *Bacteroidetes*, *Firmicutes*, and *Prevotella* spp. [29,56,57]. Furthermore, gut microbiota composition changes with disease progression, and correlates with increased disease duration and seropositivity [58]. Adherence to a Mediterranean diet seems to restore this dysbiosis, leading to reduced CRP levels and lower disease activity in patients with established RA [59].

The need of the hour is to define the nutritional state of every individual, as was evidenced from the Swedish epidemiological investigation of RA (EIRA) where negative association between a Mediterranean diet and RA risk was restricted only in men with RF-positive RA [49]. Advances in high-throughput omics technologies in combination with advanced computational algorithms will help us understand why individuals respond differently to the same dietary interventions. Furthermore, we can use omics technologies to identify the effects of each dietary food intervention on an individual’s health, thereby allowing us to explore the possibility of utilizing precision nutrition to maximize our window of opportunity for RA prevention.

## 9. Randomized Controlled Trials to Prevent RA in Genetically Predisposed Individuals

There are considerations regarding clinical trial recruitment for individuals with genetic predisposition, specifically those with first-degree relatives who have RA. Most RA prevention trials aim to recruit individuals with autoantibodies [60], but little consideration is made regarding predisposing genetic risk factors. It is assumed that much of genetic risk associated with RA development is captured through the development of antibodies [61]; however, it remains unclear if relatives of RA patients with ACPA positivity have the same risk of arthritis as individuals with ACPA positivity who do not have a relative with RA. Many RA prevention trials also enroll based on the presence of arthralgia (with ACPA) [62]. We have previously reported Indigenous FDR have increased prevalence of self-reported joint symptoms compared to non-FDR [63]. This finding is not well understood; whether this phenomenon is mediated by the same genetic loci that lead to RA risk, or because of familial proximity and awareness of RA itself, remains a key question to address. Importantly, relief from arthritis symptoms is an important facilitator for the recruitment of participants into prevention trials [39], but this must be balanced as an inclusion criterion given modest association between arthralgia and arthritis onset. Selection of the study intervention treatment also needs to be weighed against a variety of important considerations. Most RA prevention trials have deployed medications used in established RA to try and “shift” the window of opportunity in individuals without clinically detectable arthritis. Rituximab was shown to delay RA onset [64], but was associated with a significant adverse event rate in the active treatment group, and this may inform the results of other ongoing prevention trials including Hydroxychloroquine and Abatacept [65]. However, as the risk of RA in otherwise well individuals increases, in part due to genetic predisposition, the tolerability of higher-risk interventions may also increase.

With these considerations, our research group is planning a prospective clinical trial to investigate the use of curcumin, omega-3, and vitamin D (COD) to modulate RA-associated autoimmunity in Indigenous peoples. Murine models of arthritis suggest that COD can modulate onset and disease activity of arthritis, which supports the biological plausibility of this intervention [55]. Using a randomized, blinded cross-over design has the advantage of ensuring all participants will receive the active intervention treatment, with each individual serving as their own control, which also enhances the statistical power of the analysis [66]. We aim to enroll ACPA-seropositive individuals, regardless of symptoms or family history of RA, and have powered the trial to compare ACPA levels after 3 months or treatment, rather than targeting disease prevention, which will reduce the study burden. Our intervention has distinct advantages, perceptually, given it is derived naturally, is a Health Canada (HC) approved, over-the-counter-supplement and is dosed orally once daily.

Historical and contemporary research involving Indigenous peoples, including First Nations people in Canada, has often been unethical, harmful, and for the benefit of the researcher or institution [67]. To overcome the harms of the past and place power and control of the research process in the hands of Indigenous peoples, development of specific research guidelines and policies for RCTs involving Indigenous peoples are crucial. Beyond a biomedical focus, Indigenous health research needs a holistic research focus taking into account physical, mental, emotional and spiritual health. Research involving First Nations peoples must be culturally appropriate, respectful, empowering, needs based, productive and bridge capacity building. It has been noted that RCTs that included Indigenous voices through qualitative interviews were successful in better understanding the participant view of the study and collected additional information to apply that directly to future RCTs for improvement of the study processes [22]. In the spirit of working respectfully with First Nations, the inclusion of Indigenous voices is an important aspect of this work. Table 1 highlights quotes from Indigenous participants who have contributed to our understanding of prevention of RA within First Nations populations.

## 10. Conclusions

Studies from multiple populations have shown that there exists a lengthy prodromal period of subclinical autoimmunity prior to the onset of clinically detectable joint disease in RA. This prodromal period can be as short as a few weeks or span several years. These studies, including our own, which were undertaken in a predisposed Indigenous North American population, have shown that genetic predisposition plays a particularly important role in amplifying and maturing the autoimmune responses, ultimately rendering them pathological. Conversely, the detection of RA-associated autoantibodies, such as ACPA and RF, in an otherwise unaffected individual does not accurately predict future disease in a substantial proportion of individuals. Indeed, we have shown that many individuals who at one time are seropositive for ACPA and/or RF become seronegative for these autoantibodies over time, particularly if they are detected at low levels. Additionally, these autoantibodies may reappear at a later time. In the timeless words of the Nobel Laureate Niels Bohr “Prediction is very difficult, especially if it’s about the future”!

Notwithstanding our incomplete understanding of preclinical RA, and the availability of a robust risk model, this prodromal period of RA autoimmunity has raised the tantalizing prospect of developing strategies that may prevent the onset of RA. This is a progressive lifelong disorder that currently can only be suppressed, but not cured. A spectrum of potential interventions is being evaluated in at-risk cohorts around the world. This spectrum includes pharmacologic therapies that are available to treat established RA, and a variety of lifestyle modifications, which, if applied early enough, may have a substantial impact on the risk of future RA.

How do we convince unaffected individuals that the risks of a particular prevention strategy outweigh those of developing future RA? The understanding and interpretation of these risks, and how they can be balanced, will likely vary considerably depending on familial and cultural factors. An individual from a close-knit community who is surrounded by multiple family members with RA will almost certainly have a different perspective than one who has barely heard of this disease, even if both individuals have detectable RA autoantibodies. Moreover, the specific intervention proposed is an important factor. The perceptions around taking a daily pill such as hydroxychloroquine will differ from taking injections of a biologic such as abatacept, or a single infusion of rituximab. Lifestyle modifications such as smoking cessation or major dietary changes are notoriously difficult to achieve, even in highly motivated individuals.

Our studies of the at-risk family members of First Nations RA patients have led us to conclude that the use of readily available immunomodulatory/anti-inflammatory nutritional supplements, achieves a good balance of potential efficacy, safety, and acceptability for our study population. Supplements of curcumin, omega-3 fatty acids, and vitamin D, used in combination, have the potential to modulate the progression of preclinical RA autoimmunity based on both animal models and limited human studies. The well-established safety record and low cost of these supplements are particularly appealing, although the efficacy in modulating preclinical RA autoimmunity remains to be demonstrated in informative clinical trials. As such, the design of these trials is challenging from multiple perspectives, particularly the identification and recruitment of an appropriate study population, and the development of surrogate immunological endpoints that can serve as meaningful outcome measures. International consortia have been assembled to address these important challenges [68].

## Figures and Tables

**Table 1:** Indigenous (First Nations) peoples voice, in response to questions about RA “risk” and prevention activities (Source: Local focus groups held in several First Nations communities).

*Prevention/Thinking about RA* P: No. I’ve a lot of other stuff to … think about. P: I don’t like pills neither… like even if I did get a prescription, it’d probably just sit in my cupboard at home anyways … take it as I need it. Same thing with ah pills for arthritis you know (..) It probably sit in the cupboard and take it when I need it when I feel pain. P6: So I was thinking. Like I’m young and I want to have kids someday. It’s just, don’t know, it just makes me think, what everybody is saying so … And that’s another thing that ah … that science will have to … to look at. Like that … which lies dormant in our bodies you know I mean coming from a family of ah, a family history of rheumatoid arthritis … I think it’s important for science to … to, to look into it.
*Age as a Factor in Thinking about Prevention* P3: If I had known that I’d, it, I probably would-a, I probably would-a stopped smoking. If I had known that. But, if I was younger and if I had symptoms as a teenager I’d probably wouldn’t of. I think, I think that the age has a lot to do with that. If I was 17 years old and was smoking, and somebody told me, “Hey, If you keep smoking you’re gonna get arthritis. I would think right away, “That’s not true. I’m gonna keep smoking”.
*Family Considerations* P2: Because I feel for my babies, to have arthritis too. My kids.
*Perceptions of Medication* P1: Well if the doctor knows that it had prevented. He’s the doctor, certified doctor I would trust Dr. _ if he told me that. “Take this pills you won’t have that”. I believe I would take them because of him, ‘cause I know he went through people who had taken those pills and you knew the results, rather than some doctor coming in here and giving me these pills the doctor I don’t know.
*Evidence* P1: You know, I guess that’s where you begin to question the, ah scientific ah researches that are, that are happening. You know you’d think by now they, they would be able to clearly define these things that we’re wondering about. That’s my view on that. I guess the only thing that can, can convince me is like when I get to that stage. The symptoms are symptoms but until it actually happens I guess that’s when I would believe that I have rheumatoid arthritis.
*Participation in Research* P (male): I would, I would buy into it if it would lead to the prevention of the disease itself, you know being a young guy myself going onto 50 60 years old you know, tracking my record like that leading to different … growth; treatments whatever … I would like to help, in any way I can. Just because I’ve seen like, it hurt my family you know like, like what it did to my family. I wouldn’t want it to happen to anybody else if there’s a possibility that I could do something about it…
*Traditional/Cultural Knowledge* P2: Can I tell you something I was telling my sister about that when _ called me. like my grandfather … ah I’m off track here, ah you know he was a trapper, he was on a trap-line and he … often had his feet wet and he was always wet and in the spring, in the fall, in the winter eh and I was just a little girl and I remember he was talking to us telling us it was so important that we had to get away from his way of life and we would never ever have arthritis not that he was suffering at that time already and he was probably I would say 45 around there maybe. And why-why did he so why did he … he had this bad well there was a Cree word for that ‘eh and that’s what it was arthritis. And there’s a Cree word for that.
